# A multi-study exploratory investigation into choking under pressure frequency, self-compassion, and depressive symptoms in athletes

**DOI:** 10.3389/fpsyg.2026.1788534

**Published:** 2026-10-29

**Authors:** Christopher Mesagno, Andrew Jago, Adwoah A. Hammond, Scott Benedict, Matthew A. Goodyear, Jake Gibson

**Affiliations:** Institute for Health and Sport, Victoria University, Melbourne, VIC, Australia

**Keywords:** choking frequency, mental health, performance failure, self-care, sport

## Abstract

This exploratory, quantitative multi-study paper aims to determine if self-compassion moderates the relationship between choking under pressure (i.e., choking) frequency and depressive symptoms. In Study 1, 213 athletes who currently competed in, or recently retired from, competitive sport agreed to complete a survey that included demographics information, choking frequency in the last year of sport participation, self-compassion scale, and depressive symptoms scale, questions. Moderation analysis found no significant moderation effects for self-compassion on choking frequency in the last year and depressive symptoms, which was likely the result of confounding variables in the Study 1 design. Thus, using an independent sample of 303 athletes, a follow-up study (i.e., Study 2) was conducted. Methodological changes included controlling for confounding variables present in Study 1, alongside choking frequency measured within the last 3 months and 1 month. Study 2 findings indicated that a positive relationship between choking frequency and depressive symptoms exists. Furthermore, a significant moderation effect occurred for self-compassion on choking frequency within the last 3 months and depressive symptoms in the overall sample. Athletes with low self-compassion showed stronger positive associations between choking frequency within the last 3 months and depressive symptoms, while those with higher self-compassion exhibited resilience to the negative effects of choking. This multi-study paper was the first study, to date, to identify that self-compassion may moderate the association between choking frequency and adverse mental health outcomes, such that lower self-compassion may heighten vulnerability, whereas higher self-compassion may provide a potential protective buffer, to adverse mental health consequences following choking experiences.

## Introduction

Choking under pressure (i.e., choking) in sport is a phenomenon that affects athletes of all experience levels and within all sports. Defined as “an acute and considerable decrease in skill execution and performance when self-expected standards are normally achievable, and which is the result of increased anxiety under perceived pressure” (Mesagno and Hill, 2013, p. 273), choking is experienced through rare (i.e., acute) or recurrent (i.e., chronic) occasions (Mesagno et al., 2024), which may lead to harmful psychological repercussions for athletes (Hill et al., 2019). Research on the negative psychological impact after experiencing choking has only just begun, and researchers are now asking questions related to why mental health issues exist due to choking.

Hill et al. (2009) first proposed that choking could adversely affect an athlete’s psychological well-being, subsequently finding that athletes who often experience choking have low self-worth and heightened emotional distress. Suicidal ideation is another psychological consequence (Hill et al., 2019), with recent quantitative research identifying that 7.1% of athletes reported suicidal thoughts as a consequence of choking (Mesagno et al., 2024). Although research indicates that mental health issues (e.g., depression) may arise from choking incidences (Mesagno et al., 2024), the potential for choking to be associated with depressive symptoms remains ambiguous with limited research investigations attempting to understand variables that may exacerbate depressive symptoms as a result of choking. The mental health impacts of choking may be exacerbated when chronic choking occurs since more frequent choking experiences may lead to negative feelings toward playing sport and thoughts about dropping out of sport altogether (i.e., retiring; Mesagno et al., 2024). Nevertheless, performance failure, which is an athlete’s inability to execute a specific skill or performance at their normal standard during a competition or practice, has been positively correlated with depressive symptoms (e.g., Davis et al., 2008; Hammond et al., 2013). In fact, Jones and Sheffield (2007) found that athletes reported higher negative mood states, such as depression, up to 6 days after performance failure (i.e., loss), than those with successful performance (i.e., win) in competition. Since choking is a more extreme type of performance failure under pressure, similarities between choking and performance failure may exist (Mesagno et al., 2024).

### Performance failure and depressive symptoms

Since there are winners and losers, performance failure is an unavoidable part of sport, but an athlete’s responses to failure can influence their mental health. Following performance failure, athletes frequently engage in self-evaluation and self-criticism (Kowalski and Duckham, 2014), which may serve as a motivational mechanism to improve performance. When excessively negative self-assessment occurs, however, it may lead to irrational self-beliefs, particularly in evaluative contexts, adversely affecting an athlete’s mental health (Popov et al., 2015). Excessive self-criticism has been positively linked to depressive symptoms among athletes (Oliveira et al., 2022; Walton et al., 2020). Furthermore, athletes may use negative attribution styles (e.g., maladaptive explanations) after performance failure, which may lead athletes to internalize failure as a personal deficiency, increasing possible mental health risk (Nixdorf et al., 2016).

### Self-compassion as a buffer for performance failure

Self-compassion has been explored to increase an athlete’s ability to cope with incidences of performance failure in sport (Killham et al., 2018; Mosewich et al., 2011) since it may act as a buffer to help decrease potential mental health risks. Self-compassion, which is the ability to show kindness to oneself (Neff, 2003), mitigates depressive symptoms and maladaptive behaviors that result from performance failure (Reis et al., 2015). However, little is known about how self-compassion links to the psychological consequences of choking. Self-compassion consists of three interconnected components: self-care versus self-judgment, where suffering is alleviated through self-care practices and concern rather than self-criticism (Ceccarelli et al., 2019; Neff, 2003); mindfulness versus overidentification refers to an individual having an open view of one’s emotions without overidentifying with them or avoiding them (Ceccarelli et al., 2019; Neff, 2003); common humanity versus isolation, which entails accepting failure as a common human, rather than an isolated, experience (Ceccarelli et al., 2019; Neff, 2003). Self-compassion is negatively correlated with guilt, shame, negative affect, and avoidance, whereas positive sport performance evaluations and increased psychological wellbeing show a positive association with self-compassion (Killham et al., 2018; Mosewich et al., 2013). Mosewich et al. (2013), however, failed to include male athletes in their sample, which brings limitations and questions the generalizability of results. Furthermore, Wilson et al. (2019) suggest that self-compassion assists athletes in managing performance failures and high-pressure situations by allowing them to draw strength from previous successes, subsequently counteracting any negative thinking and reducing self-criticism.

Elite-level athletes find it challenging to adopt self-compassion practices due to fears of stigmatization and appearing to lack mental toughness (Ceccarelli et al., 2019; Sutherland et al., 2014). Therefore, elite athletes are less likely to embrace self-compassion techniques than lower-level (i.e., recreational/club representatives) athletes (Poucher et al., 2021; Walton et al., 2020). Competitive environments, an increased capacity to be self-critical, and a desire to strive for a higher social rank (i.e., comparison to others on a hierarchy of athletic status) are common factors in high-level sports that may reduce the likelihood of athletes adopting self-compassion practices (Hardy et al., 2017; Poucher et al., 2021; Walton et al., 2020). A high-level athlete’s disposition to reject self-compassion and be self-critical may be connected with striving for a desired social rank, where this pursuit may increase the onset of depressive symptoms (Killham et al., 2018). This limited self-compassion may be exacerbated when choking frequency is elevated, considering the essence of being an elite athlete is the ability to perform well under pressure and the threat of being labeled a “chronic choker” may be devastating to an athlete’s identity (Mesagno et al., 2011). Thus, it would be even more psychologically damaging for elite athletes to deal with multiple choking experiences, which may lead to increased mental health issues without employing higher self-compassion practices. From a moderation analysis perspective, these contextual and dispositional factors suggest that self-compassion may function as a protective factor that alters the strength of the relationship between choking experiences and depressive symptoms.

### Current multi-study paper

Given the limited aforementioned research, athletes who experience choking often (i.e., higher choking frequency) may be less inclined to use self-compassionate practices, which may lead to increased incidences of depressive symptoms, since they have more self-judgment and are unable to accept the negative performance outcomes of choking under pressure well. Thus, the purpose of the current exploratory, quantitative multi-study research was to determine if self-compassion moderates the relationship between choking frequency and depressive symptoms.

## Study 1

### Method

#### Participants

Two hundred and thirteen participants who currently compete in (*n* = 88; 53%), or recently retired from (*n* = 77; 47%), competitive sport agreed to participate in Study 1. Participants were aged from 18 to 76 (M*_*age*_* = 33.82; *SD_*age*_* = 12.25) and competed in a total of 32 competitive sports. Experience level in competitive sports ranged from less than 6 months to over 30 years. The sample included 75 club-level (i.e., hereafter known as low-level) athletes, 41 state-level athletes, 40 collegiate/national, and nine international/Olympic-level (i.e., hereafter known collectively as high-level) athletes.

*A priori* power analysis was performed using G*Power version 3.1.9.7 (Faul et al., 2007) to determine the minimal sample size required for the study. The required sample size to achieve 80% power for detecting a medium effect with a significance level of 0.05 was 84. Therefore, the generated sample size of 165 was sufficient.

### Materials

#### Demographics questionnaire

The demographic questionnaire was identical to Mesagno et al. (2024), which included age, gender, involvement in competitive sport, and frequency of choking in sport, but excluded questions related to psychological, behavioral, suicidal ideation, and maladaptive behavior linked to choking. Within the choking section, we used Mesagno et al.’s choking definition/description: Choking under pressure in sports can be seen as a significant decline in athletic performance at a time of increased nervousness. We also used Mesagno et al.’s choking frequency measure, How many times in the last year of playing your sport have you experienced choking?, where answers were measured from 0 to 200 choking experiences in the last year.

#### Centre for epidemiologic studies depression scale (CES-D)

The CES-D assesses the depressive symptoms experienced across the previous week (Radloff, 1977). Participants answer 20 questions with responses consisting of a 4-point rating scale ranging from 0 (rarely or none of the time) to 3 (most or all of the time). Participant scores range from 0 to 60, with higher scores indicating higher depressive symptoms. Items 4, 8, 12 and 16 require reverse scoring before computing a total CES-D score. Hautzinger and Bailer (2003) indicated the CES-D as being psychometrically sound, revealing a Cronbach’s alpha of 0.89. Reliability analysis for the current sample was 0.89, indicating strong internal consistency (Fleiss, 1986).

#### Self-compassion scale short form (SCS-SF)

Self-compassion was measured using the SCS-SF, a 12-item self-report instrument that measures an adult’s capacity to feel self-compassion. The self-compassion scores are based on an individual’s ability to feel warmth, connectedness, and concern for their respective struggles (Raes et al., 2011). The Likert-type scale ranges from 0 (almost never) to 5 (almost always). Items 1, 4, 8, 9, 11 and 12 need to be reversed before computing a total mean score for self-compassion. A higher score indicates higher levels of self-compassion. Generally, scores between 1.0 and 2.49 are considered low, scores between 2.5 and 3.5 are moderate, and those between 3.51 and 5.0 are high (Raes et al., 2011). Overall self-compassion scores indicated strong internal consistency with a Cronbach’s alpha of ≥0.86 (Raes et al., 2011). Cronbach’s alpha for the current sample was 0.86, indicating strong and satisfactory internal consistency (Fleiss, 1986).

### Procedure

After University Human Research Ethics was obtained, individuals were recruited directly or via an online social media advertising campaign through social media posts and our sport networks. A Qualtrics link to the survey (interested researchers can email the first author for survey requests), including a plain language statement informing participants of their ethical rights (e.g., confidentiality, right to withdraw), was attached to the social media posts and sport networks messages.

Upon accessing the survey link, participants provided informed consent by ticking “yes,” which was next to the consent to participate box, before commencing the survey. Participants were first provided with the demographic questionnaire, followed by the CES-D (Radloff, 1977), and the SCS-SF (Raes et al., 2011). Participants took between 7 and 13 min to complete the survey. After the survey, participants were thanked for their contribution and provided the primary researcher’s contact details in the unlikely event they were distressed from any parts of the survey.

### Statistical analysis

Data collected from Qualtrics (Qualtrics, Provo, UT) was exported into the Statistical Package for Social Sciences (SPSS Statistics Version 29; IBM SPSS Inc., Chicago, IL) for analysis. Reverse scoring for necessary items was completed prior to analyses. A total depressive symptoms score (i.e., total CES-D) and a total mean self-compassion score (i.e., total mean SCS-SF) were calculated. Choking frequency was measured using self-report questions (as described above). A positive skew was observed in the choking frequency and total depressive symptoms variables and rectified through logarithmic transformation prior to analysis. Although normality remained imperfect, the sample sizes were considered sufficient for the regression analyses. Univariate outliers remained in the data set as Cook’s distance was not violated.

Demographic variables were analysed and summarized using descriptive statistics. Using PROCESS macro (Model 1; Hayes, 2022), separate cross-sectional moderation analyses were performed to assess if total mean self-compassion altered the relationship between choking frequency and total depressive symptoms in the overall, and high-level athletes, sample. The primary analysis was specified in advance whereas the secondary analysis related to high-level athletes was exploratory. Analyses were conducted using listwise deletion, such that participants with missing data on any variable required for a given analysis were excluded from that analysis.

## Results

The sample consisted of 75 low-level (i.e., club-level representatives) and 90 high-level (i.e., state, collegiate, national, or international/Olympic) athletes. Descriptive statistics on the choking frequency, total depressive symptoms (prior to logarithmic transformation), and total mean self-compassion on the overall sample are presented in Table 1.

**TABLE 1:** Descriptive scores for depressive symptoms (total CES-D), self-compassion (total mean SCS-SF), and choking frequency in the last year of competitive sport for the overall sample in Study 1.

Scale	*M*	*SD*
Total CES-D	13.23	9.03
Total mean SCS-SF	3.15	0.72
Choking frequency	18.25	23.05

### Assumption testing for primary statistical analyses

The assumption of normality was violated. As the sample size in each analysis was greater than 30 participants, this need not be rectified (Pallant, 2016). All other assumptions for each statistical analysis were met.

### Moderation analysis of overall sample (*n* = 97)

The overall model was statistically significant, *F*(3, 93) = 11.27, *p* < 0.001, and accounted for approximately 26.7% of the variance in depressive symptoms (R^2^ = 0.267). Choking frequency within the last year did not significantly predict depressive symptoms (*b* = −0.19, SE = 0.28, *t*(93) = −0.69, *p* > 0.10, 95% CI [−0.75, 0.36]). Self-compassion was a significant negative predictor of depressive symptoms (*b* = −0.31, SE = 0.11, *t*(93) = −2.73, *p* = 0.008, 95% CI [−0.54, −0.09]), indicating that higher self-compassion was associated with fewer depressive symptoms. The interaction term between self-compassion and choking frequency was not significant, *b* = 0.07, SE = 0.09, *t*(93) = 0.77, *p* > 0.10, 95% CI [−0.11, 0.25]. This indicates that choking frequency within the last year did not moderate the association between self-compassion and depressive symptoms. The change in R^2^ attributable to the interaction term was negligible (ΔR^2^ = 0.005, *F* = 0.61, *p* = 0.438).

### Moderation analysis on high-level athletes (*n* = 53)

For the high-level athletes, the overall model explained a significant proportion of variance in depressive symptoms, *F*(3,49) = 8.58, *p* < 0.001, R^2^ = 0.34. Choking frequency did not significantly predict depressive symptoms (*b* = −0.11, SE = 0.10, *t*(49) = −1.04, *p* > 0.10, 95% CI [−0.31, 0.10]). The moderation analysis revealed that higher self-compassion predicted lower depressive symptoms, *b* = −0.26, SE = 0.05, *t*(49) = −4.90, *p* < 0.001, 95% CI [−0.37, −0.15], however, self-compassion was non-significant when moderating the relationship between the choking frequency and depressive symptoms, *b* = 0.01, SE = 0.13, *t*(49) = 0.10, *p* > 0.10, 95% CI [−0.25, 0.27].

## Discussion

Results from Study 1 indicated that self-compassion does not moderate the relationship between choking frequency and depressive symptoms for the overall or high-level athletes samples, although these results could be due to a number of constraints within the research design. For example, participants reported their choking frequency within the past year of competitive sport, while depressive symptoms were assessed during the previous week. This may have limited the ability to associate the two together due to discrepancies between the timelines. Additionally, both current and retired athletes were collectively a part of the sample. The ability for a retired athlete to recall information may have been impacted since they may have been out of sport longer and contributed to the inaccuracies between choking frequency and depressive symptoms. For these reasons, a second study was conducted with these confounding variables controlled. Thus, Study 2 was a follow-up exploratory study to Study 1. Specifically, Study 2 differed in that only currently competing athletes participated and choking frequency was limited to the past 3 months and within the last month. Further, the instructions for completing the questionnaires were altered slightly so athletes reflected on past choking experiences when answering questions, to create a clearer link between self-compassion, depressive symptoms, and choking.

## Study 2

### Method

#### Participants

A total of 303 participants, aged from 18 to 80 years old (*M*_*age*_ = 34.68, *SD* = 12.23) who currently competed in a range of competitive sports, voluntarily participated in Study 2. The number of years athletes had been engaged in competitive sports ranged from 1 to 30 years (*M_*exp*_* = 12.6, *SD* = 8.96) with the sample consisting of 177 (58.4%) low-level (i.e., club) and 126 (41.6%) high-level (i.e., state/county, collegiate, national, international/Olympic) athletes.

### Materials

#### Demographics questionnaire

The demographic questionnaire included similar questions to Study 1 with the exception that we added two extra questions relating to choking frequency: During the past 3 months, approximately how many times have you experienced choking under pressure while playing your main sport? During the past month, approximately how many times have you experienced choking under pressure while playing your main sport?

#### Centre for epidemiologic studies depression scale–revised (CESD-R)

We used the CESD-R (Eaton et al., 2004), an updated version of the CES-D, to measure depressive symptoms. The scale consists of 20 items, each rated on a 5-point Likert scale with five response options ranging from not at all/less than 1 day, 1–2 days, 3–4 days, 5–7 days, or nearly every day for 2 weeks. Participants indicate how often they have experienced the symptoms over the past week, with total scores ranging from 0 to 80. Higher scores indicate higher depressive symptoms. Van Dam and Earleywine (2011) found the CESD-R to have good psychometric properties which included high internal consistency, strong factor loadings and validity with anxiety, schizotypy, and positive and negative affect. Reliability analysis of the current sample was 0.77. A Cronbach’s alpha value above 0.70 is considered adequate for research purposes (Peterson, 1994), therefore the current sample demonstrated acceptable internal consistency.

#### Self-compassion scale short form (SCS-SF)

Self-compassion was measured using the SCS-SF, identical to Study 1 of this manuscript.

### Procedure

Identical procedures to Study 1 of this manuscript were used with the following exceptions: First, a paid online data collection platform Prolific,^1^ which is a high quality and reliable data collection platform (Douglas et al., 2023), was also used to recruit participants. Each participant associated with Prolific was reimbursed AU$10 for their time. Second, the instructions for the CESD-R and SCS-SF were changed to emulate more choking specific situations since Study 1 participants completed the survey with the instructions having temporal inconsistencies (e.g., choking frequency was measured the past year, while depressive symptoms were assessed in the previous week). This scale instruction adjustment may help participants recall the cognitions associated with the performance experiences more vividly. Instructions wording was identical to Study 1 outside of the choking-specific changes. The instructions for the CESD-R read, “For each statement, please reflect on a recent choking under pressure experience and indicate how often you have felt each statement in the past week or so after the choking under pressure event.” The instructions for the SCS-SF read, “Please reflect on a recent choking under pressure experience and read each statement carefully before answering. Please indicate how often you behave in the stated manner in the week after a recent choking under pressure event.” It took all participants between 6 and 12 min (an average of 8.81 min) to complete the survey.

### Statistical analysis

Since Study 2 controlled for the confounding variables identified in Study 1, the moderation effects of self-compassion on choking frequency and depressive symptoms at closer temporal survey points were analysed. Choking frequency was entered as the independent variable (X), depressive symptoms (CESD-R Total) as the dependent variable (Y), and self-compassion (total mean SCS-SF) as the moderator (W). Several moderation analyses were conducted using the PROCESS macro (Model 1; Hayes, 2022) to examine whether choking frequency within the last 3 months and 1 month, respectively, moderated the relationship between self-compassion and depressive symptoms. Separate moderation analyses were conducted for the overall sample and different skill (i.e., low and high) levels, respectively. Similar assumption and normality testing, and listwise deletion as Study 1 was conducted prior to statistical analyses. The primary analyses related to choking frequency (at any time point) were determined in advance, whereas the secondary sample analysis related to different skills levels was exploratory. All continuous variables were mean-centered prior to analysis.

## Results

Of the 303 total sample, 177 were low-level (i.e., club-level representatives) and 126 high-level (i.e., state/county, collegiate, national, or international/Olympic) athletes.

### Moderation analysis of overall sample - choking frequency within the last 3 months (*n* = 171)

The overall model was statistically significant, *F*(3, 167) = 21.39, *p* < 0.001, and accounted for approximately 27.8% of the variance in depressive symptoms (R^2^ = 0.278). Choking frequency in the last 3 months significantly predicted depressive symptoms (*b* = 0.40, SE = 0.15, *t*(167) = 2.61, *p* = 0.010, 95% CI [0.10, 0.69]). This indicates that more frequent choking experiences within the last 3 months were associated with higher levels of depressive symptoms. Self-compassion was also a significant negative predictor of depressive symptoms (*b* = −6.05, SE = 0.85, *t*(167) = −7.12, *p* < 0.001, 95% CI [−7.73, −4.37]), indicating that higher self-compassion was associated with fewer depressive symptoms. The interaction between choking frequency and self-compassion was statistically significant (*b* = −0.54, SE = 0.24, *t*(167) = −2.19, *p* = 0.030, 95% CI [−1.02, −0.05]), suggesting that the relationship between choking frequency within the last 3 months and depressive symptoms depended on levels of self-compassion. The change in explained variance due to the interaction was ΔR^2^ = 0.021, *F*(1, 167) = 4.80, *p* = 0.030, indicating that the inclusion of the interaction term improved the model’s explanatory power.

Conditional effects were examined at one standard deviation below, at, and one standard deviation above, the mean of self-compassion. At low self-compassion (−0.63 SD), the relationship between choking frequency and depression was positive and significant (*b* = 0.73, SE = 0.21, *t*(167) = 3.46, *p* = 0.001, 95% CI [0.31, 1.15]). At average self-compassion, the relationship remained significant but weaker (*b* = 0.40, SE = 0.15, *t*(167) = 2.61, *p* = 0.010, 95% CI [0.10, 0.69]). At high self-compassion (+0.63 SD), the relationship was nonsignificant (*b* = 0.06, SE = 0.22, *t*(167) = 0.27, *p* = 0.788, 95% CI [−0.37, 0.49]). The Johnson–Neyman test identified a significant region of moderation below a self-compassion score of 0.16, indicating that choking frequency within the last 3 months was positively associated with depressive symptoms only when self-compassion was below this value.

### Moderation analysis of low-level athletes - choking frequency within the last 3 months (*n* = 105)

The overall model was significant, *F*(3, 101) = 10.34, *p* < 0.001, accounting for approximately 23% of the variance in depressive symptoms (R^2^ = 0.23). Choking frequency within the last 3 months was also a significant positive predictor of depressive symptoms, *b* = 0.42, SE = 0.20, *t*(101) = 2.13, *p* = 0.036, 95% CI [0.03, 0.81]. There was a significant main effect of self-compassion, such that higher self-compassion was associated with lower depressive symptoms, *b* = −5.27, SE = 1.05, *t*(101) = −5.01, *p* < 0.001, 95% CI [−7.36, −3.18]. The interaction was not significant (although it approached significance), indicating that the association between choking frequency and depressive symptoms did not vary as a function of self-compassion, *b* = −0.70, SE = 0.37, *t*(101) = −1.88, *p* = 0.063, 95% CI [−1.43, 0.04], contributing an additional 2.7% of explained variance, ΔR^2^ = 0.027, *F*(1, 101) = 3.52, *p* = 0.063.

### Moderation analysis of high-level athletes - choking frequency within the last 3 months (*n* = 65)

The overall model was significant, *F*(3, 61) = 11.32, *p* < 0.001, explaining approximately 36% of the variance in depressive symptoms (R^2^ = 0.36). Choking frequency within the last 3 months was not a significant predictor, *b* = 0.31, SE = 0.24, *t*(61) = 1.31, *p* = 0.20, 95% CI [−0.17, 0.79]. In contrast, self-compassion emerged as a significant negative predictor of depressive symptoms, *b* = −8.20, SE = 1.57, *t*(61) = −5.23, *p* < 0.001, 95% CI [−11.33, −5.07], indicating that higher self-compassion was associated with lower depressive symptoms. The interaction between self-compassion and choking frequency was not significant, *b* = −0.29, SE = 0.36, *t*(61) = −0.82, *p* = 0.42, 95% CI [−1.01, 0.42], and did not account for additional variance in depressive symptoms, ΔR^2^ = 0.007, *F*(1, 61) = 0.67, *p* = 0.42.

### Moderation analysis of overall sample - choking frequency within the last month (*n* = 86)

The overall model was significant, *F*(3, 82) = 17.50, *p* < 0.001, explaining 39.0% of the variance in depressive symptoms (R^2^ = 0.390, *R* = 0.625). Choking frequency within the last month positively and significantly predicted depressive symptoms (*b* = 1.90, SE = 0.64, *t*(82) = 2.95, *p* = 0.004, 95% CI [0.62, 3.18]), which indicates that the more frequent choking experiences within the last month, the higher levels of depressive symptoms. Self-compassion was also a significant negative predictor of depressive symptoms (*b* = −7.45, SE = 1.16, *t*(82) = −6.41, *p* < 0.001, 95% CI [−9.76, −5.13]), indicating that higher self-compassion was associated with fewer depressive symptoms. The interaction term between self-compassion and choking frequency was not significant, *b* = −0.57, SE = 0.89, *t*(82) = −0.64, *p* = 0.524, 95% CI [−2.33, 1.20]. This indicates that self-compassion did not moderate the association between choking frequency within the last month and depressive symptoms for the overall sample. The change in R^2^ attributable to the interaction term was negligible (ΔR^2^ = 0.003, *F* = 0.41, *p* = 0.524).

### Moderation analysis of low-level athletes - choking frequency within the last month (*n* = 50)

The overall model significantly predicted depressive symptoms, *F*(3, 46) = 7.05, *p* = 0.0005, explaining 31.5% of the variance (R^2^ = 0.315). Choking frequency was a significant, positive predictor, *b* = 2.06, SE = 0.99, *t*(46) = 2.08, *p* = 0.043, 95% CI [0.065, 4.048] of depressive symptoms. Self-compassion was also significantly, negatively, *b* = −7.45, SE = 1.16, *t*(46) = −4.15, *p* = < 0.001, 95% CI [−10.95, −3.80], associated with depressive symptoms. The interaction between self-compassion and choking frequency was not significant, *b* = −1.07, SE = 2.25, *t*(46) = −0.48, *p* = 0.635, 95% CI [−5.56, 3.45]. This indicates that, for low-level athletes, self-compassion did not moderate the relationship between choking frequency within the last month and depressive symptoms. Because the interaction was non-significant, the effect of choking frequency on depressive symptoms did not differ across self-compassion.

### Moderation analysis of high-level athletes - choking frequency within the last month (*n* = 36)

The model explained 53.7% of the variance in depressive symptoms, R^2^ = 0.537, and was statistically significant, *F*(3, 32) = 12.39, *p* < 0.001. Choking frequency was a significant predictor, *b* = 1.68, SE = 0.81, *t*(32) = 2.08, *p* = 0.045, 95% CI [.037, 3.33], with higher choking frequency associated with higher depressive symptoms in high-level athletes. Self-compassion significantly predicted depressive symptoms, *b* = −8.27, SE = 1.86, *t*(32) = −4.44, *p* < 0.001, 95% CI [−12.06, −4.47], with higher self-compassion associated with lower depressive symptoms in high-level athletes. The interaction term was not significant, *b* = −0.42, SE = 0.95, *t*(32) = −0.44, *p* = 0.663, 95% CI [−2.36, 1.52] indicating that self-compassion did not moderate the effect of choking frequency within the last month on depressive symptoms. Thus, even though self-compassion uniquely predicted depressive symptoms in high-level athletes, this relationship did not depend on choking frequency in the last month.

## Overall discussion

To date, limited research has been conducted on the variables that may influence the relationship between choking frequency and mental health issues. This multi-study paper was the first to explore, through quantitative method, whether self-compassion moderates the relationship between choking frequency and depressive symptoms. Study 1 found that self-compassion did not moderate the relationship between choking frequency and depressive symptoms, which was likely due to confounding variables in the study design such as recruiting retired athletes and asking participants about choking frequency in the past year, rather than closer in timeline to the other psychological surveys used. Thus, Study 2 was a follow-up to Study 1, controlling for these confounding variables. Study 2 findings indicated that for the overall sample of athletes, self-compassion moderated the relationship between choking frequency and depressive symptoms only when choking was assessed over a 3-month timeframe and in the overall sample analysis, but not in any other choking frequency or skill level (see Table 2 for ease of understanding the combined results of each study). From the combined results, we discuss three main findings: the choking frequency and depressive symptoms association, self-compassion and depressive symptoms relationship, and moderating relationship self-compassion offers between choking frequency and depressive symptoms.

**TABLE 2:** Summary of results of this multi-study paper.

Study number	Study 1	Study 2	
		ΔR^2^	*P*
Choking frequency – Past 12 months, overall athlete sample	Non-significant moderation	–	
Choking frequency – Past 12 months, high-level athlete sample	Non-significant moderation	–	
Choking frequency – Past 3 months, overall athlete sample	–	0.021	0.030*
Choking frequency – Past 3 months, low-level athlete sample	–	–	0.630
Choking frequency – Past 3 months, high-level athlete sample	–	–	0.420
Choking frequency – Past month, overall athlete sample	–	–	0.524
Choking frequency – Past month, low-level athlete sample	–	–	0.635
Choking frequency – Past month, high-level athlete sample	–	–	0.663

*Significant at the 0.05 level; significant moderation.

### Choking frequency and depressive symptoms

Study 1 in this multi-study paper indicated that a non-significant (and negative) relationship existed between choking frequency in the last year and depressive symptoms. After adjusting for confounding variables in Study 2, however, a significant and positive (a reversal from Study 1) association was found for choking frequency in the last 3 months and 1 month, respectively, and depressive symptoms for all correlational analyses but the choking frequency of the high-level athletes within the last 3 months. Our results indicate that irrespective of the skill level, the higher the choking frequency, then the higher the depressive symptoms will be when asked about choking frequency within the last 3 months and 1 month, but not within the last year of competitive performance. The positive correlational results of Study 2 are consistent with previous qualitative (e.g., Hill et al., 2009, 2019) and other quantitative (Mesagno et al., 2024) research that links choking to negative psychological well-being outcomes. Even though Mesagno et al. found suicidal ideation and other negative mental health ramifications of choking, our study is the first quantitative study to provide positive correlations between choking frequency and depressive symptoms. It appears from this correlational result that chronic (i.e., recurring) choking may exacerbate depressive symptoms, which may indirectly lead to negative, maladaptive coping strategies identified in Mesagno et al. (2024) choking and mental health research.

### Self-compassion and depressive symptoms

Overall, our multi-study results support past research studies that have indicated a negative relationship exists between self-compassion and depressive symptoms. That is, all moderation analyses in Study 1 and Study 2 of this paper found that the higher self-compassion exhibited by athletes, the less depressive symptoms they might experience. These results support previous researchers (e.g., Jansen et al., 2019; Wasylkiw and Clairo, 2018) well documented findings that inverse correlations exist between self-compassion and depression (or depressive mood) in sport. Moreover, individuals with higher levels of self-compassion are less vulnerable to the experience of depressive symptoms (Han and Kim, 2023).

### Moderating role of self-compassion between choking frequency and depressive symptoms

Similar to the choking frequency and depressive symptoms results, there was no moderation effect of self-compassion on choking frequency and depressive symptoms until the confounding variables in Study 1 were addressed and the Study 2 data was analysed. Study 2 results indicated a moderation effect of self-compassion on choking frequency within the last 3 months and depressive symptoms. In the overall sample with choking frequency within the last 3 months only, individuals with low self-compassion exhibited stronger positive associations between choking frequency within the last 3 months and depressive symptoms, whereas those with higher self-compassion exhibited resilience to the negative emotional (and depressive) effects of choking frequency. The findings provide preliminary evidence that self-compassion may influence the association between choking frequency and depressive symptoms, although this effect was observed only for the 3-month timeframe in the overall sample and was not replicated across the other moderation analyses. Although the interaction between self-compassion and 3-month choking frequency was statistically significant, the interaction accounted for only 2.1% of additional variance and was not replicated in the 1-month choking frequency analysis or in analyses examining competitive level. Given the exploratory nature of the study and the number of analyses undertaken, the possibility that this finding reflects sampling variation or Type I error cannot be excluded. Non-significant moderation effects, particularly within the competitive-level analyses, should not be interpreted as evidence that moderation is necessarily absent. Accordingly, the present findings should be considered hypothesis-generating rather than confirmatory and requires replication in future research. Nevertheless, these findings indicate that self-compassion may buffer the impact of choking frequency on depressive symptoms.

One possible explanation for this result may be that after choking experiences, athletes with low self-compassion self-criticize and exhibit self-defeating cognitions (especially with increased choking frequency), which may then become more internalized and connected to low self-esteem and self-worth. In fact, Hill et al. (2019) qualitative research found that athletes who frequently ruminate on choking performances develop depressive symptoms that include low self-worth. With greater choking frequency comes more self-doubt and potentially self-hatred as a result of low self-worth, which may lead to mental health issues through depressive symptoms and even suicidal ideation (e.g., Hill et al., 2019; Mesagno et al., 2024). Although this may be a simplistic and speculative path linking low self-compassion to choking frequency and depressive symptoms, it may be a starting point to understand the cognitive mechanisms of why choking may lead to mental health issues.

Although there are limited studies to show the positive mental health benefits of self-compassion after choking, our results are similar to researchers’ results who have investigated the effects of self-compassion on performance setbacks (in which choking may be categorized). Our results add to the growing literature (e.g., Barczak and Eklund, 2020; Doorley et al., 2022; Reis et al., 2015) that higher levels of self-compassion may buffer the effects of performance setbacks and even extends beyond performance-related issues and indicates that higher self-compassion may help guard against mental health issues after negative performance (i.e., choking) occurs. In fact, Cormier et al.’s (2025) scoping review found unequivocal support for the use of self-compassion for athletes to move beyond setbacks and to help manage strong emotions. Since strong negative emotions occur in most athletes who experience choking (Mesagno et al., 2024), which are likely even more intense if choking occurs frequently (e.g., as with “chronic chokers”), having higher levels of self-compassion (or using self-compassion strategies) may help reduce depressive symptoms in the months after choking is experienced.

We initially argued that high-level athletes would be less inclined to adopt self-compassion practices (Ceccarelli et al., 2019; Sutherland et al., 2014) than lower-level athletes, since high-level athletes have increased capacity to be self-critical, and a desire to strive for a higher social rank (Hardy et al., 2017; Poucher et al., 2021; Walton et al., 2020). Contrary to these expectations, self-compassion did not moderate the impact of choking frequency on depressive symptoms among high-level athletes (within one year in Study 1, or 3 months and 1 month in Study 2), suggesting that while self-compassion is associated with better mental health, it may not alter how choking experiences are psychologically processed in elite contexts. Despite the moderation analysis of low-level athletes with choking frequency within the past 3 months approaching significance, it does not appear from our results that skill level influences the moderation link. These results should be viewed with caution though since the sample size in each of the low- and high-level athlete samples (and choking frequency within the last 3 months and 1 month) is low.

One interesting finding was the significant moderation effect for self-compassion on choking frequency within the last 3 months (Study 2) and depressive symptoms for the overall sample, whereas this significant moderation effect was not found for choking frequency within the last year (Study 1) or the last 1 month (Study 2). The moderation difference between choking frequency within the last 3 months and the last year might strengthen our argument about the confounding variables in Study 1 affected the results. Although there were likely more choking experiences for athletes over the year, the discrepancies between the timelines of the choking frequency in the last year and self-compassion and depressive symptoms scales (which asked participants to respond within the last week) may have affected the results. Furthermore, it could be argued that the choking frequency in the last 3 months (Study 2) may be long enough for athletes to process the results of the choking experiences, and for these cognitions to affect their mental health as they consider how it affects them as an athlete. The absence of moderation effects when choking frequency was assessed within the last month may indicate that self-compassion may not buffer immediate emotional responses to recent choking but rather influence how repeated performance failures are integrated over time. Further research is needed to decipher the mechanisms behind this finding.

### Limitations and future research

Choking remains as a term not clearly defined in literature (Mesagno and Hill, 2013). Although substantial efforts were made to accurately define choking in the present study via survey design, the likelihood of participants falsely conceptualizing choking remains a distinct possibility, affect the results. Another limitation is the crude measure of choking frequency we used in this multi-study research. A simple number of times a person experienced choking in the past month, 3 months, or year, may not be as robust a measure as the magnitude of the choking experienced (or severity) or a measure of how important the situation was to the athletes. This measurement also does not take into consideration differences in athletes in the number of competitions or high-pressure situation exposure frequency. Consequently, a higher reported choking frequency may partly reflect greater competitive exposure rather than a greater propensity to experience choking. These contextual characteristics, which may all influence the mental health of the athlete more strongly than the number of times choking occurs. Although research using survey-based methods are a useful initial point for understanding if there is a link among choking frequency, self-compassion, and depressive symptoms, there are limits to this approach, which could be overcome with experimental designs or better choking measurement tools developed. Another limitation of this multi-study data collection is that we altered the instructions for participants in Study 2 to make their experiences explicit to the depression and self-compassion scale they were completing, which may change the original intention of the construct measurement and psychometrics. This also meant that Study 1 and Study 2 likely were different in their memory recall, which may have altered the results, but we made this change to explore aspects of choking frequency and these constructs more closely in time. Future research could attempt to validate these instructions with other athletic samples and also determine if these changes may affect the psychometric properties of the scales.

Future research should investigate other psychological characteristics such as athletic identity, perfectionism, desire for social rank, and fear of failure as they may accentuate depressive symptoms in elite athletes (Brewer et al., 1993; Jensen et al., 2018; Walton et al., 2020). Without controlling for such factors, research is restricted in understanding the link between choking frequency and depressive symptoms in high-level athletes (e.g., Gilbert et al., 2004; Lamont-Mills and Christensen, 2006; Walton et al., 2020; Wetherall et al., 2019). Furthermore, by conducting the low- and high-level moderation analyses we implied that participants were more invested in sport and likely had a higher athletic identity (Brewer et al., 1993). We, however, did not measure athletic identity, where measuring this variable could inform researchers of how athletic identity may influence depressive symptoms after choking experiences. Finally, since this was an exploratory study to determine if there were links between these variables, it was beyond the scope of our analysis to consider important factors that may influence the associations among these variables such as gender effects or team and individual sports differences. Future researchers could investigate these links in separate follow-up studies.

## Conclusion

Taken together, these findings begin to provide necessary understanding on how self-compassion functions as a possible conditional protective factor on performance failure rather than a universal moderator. The potential buffering effect of self-compassion appears contingent on temporal proximity and cumulative exposure to choking experiences. This advances current understanding of how athletes psychologically respond to repeated performance failures and suggest that interventions targeting self-compassion may be effective when athletes are navigating longer term meaning and consequences of choking rather than its immediate aftermath. From an applied perspective, these results support the integration of self-compassion training within athlete mental health support programs.

This study is a world first that investigates associations between choking frequency, self-compassion, and depressive symptoms. Raising awareness of the ill mental health effects of choking and understanding why depressive symptoms occur after choking is needed. Ultimately, gaining a greater understanding of the harsh psychological effects of choking may help provide additional support and resources to athletes who experience choking more frequently in an attempt to reduce mental health issues or potential dropout from sport.

## Data Availability

The data supporting the conclusions of this article will be made available by the authors upon reasonable request.
